# The Role of PET in Supratentorial and Infratentorial Pediatric Brain Tumors

**DOI:** 10.3390/curroncol28040226

**Published:** 2021-07-05

**Authors:** Angelina Cistaro, Domenico Albano, Pierpaolo Alongi, Riccardo Laudicella, Daniele Antonio Pizzuto, Giuseppe Formica, Cinzia Romagnolo, Federica Stracuzzi, Viviana Frantellizzi, Arnoldo Piccardo, Natale Quartuccio

**Affiliations:** 1 Nuclear Medicine Department, Ospedali Galliera, 16128 Genova, Italy; angelinacistaro06@gmail.com (A.C.); arnoldo.piccardo@galliera.it (A.P.); 2 AIMN Pediatric Study Group, 20159 Milan, Italy; natale.quartuccio@arnascivico.it; 3 Department of Nuclear Medicine, University of Brescia and Spedali Civili Brescia, 25123 Brescia, Italy; domenico.albano@unibs.it; 4 Unit of Nuclear Medicine, Fondazione Istituto G. Giglio, 90015 Cefalù, Italy; 5 Nuclear Medicine Unit, Department of Biomedical and Dental Sciences and of Morpho-Functional Imaging, A.O.U. Policlinico G. Martino, University of Messina, 98125 Messina, Italy; riccardo.laudicella@usz.ch (R.L.); giuseppeformica23@gmail.com (G.F.); federicastra@hotmail.it (F.S.); 6 Department of Nuclear Medicine, University Hospital Zürich, 8091 Zürich, Switzerland; dapizzuto@gmail.com; 7 Nuclear Medicine Unit, Ospedali Riuniti, Torrette di Ancona, 60126 Ancona, Italy; cinzia.romagnolo@ospedaliriuniti.marche.it; 8 Department of Radiological Sciences, Oncology and Anatomical Pathology, Sapienza University of Rome, 00161 Rome, Italy; viviana.frantellizzi@uniroma1.it; 9 Nuclear Medicine Unit, A.R.N.A.S. Ospedali Civico, Di Cristina e Benfratelli, 90127 Palermo, Italy

**Keywords:** positron emission tomography, brain tumors, pediatrics

## Abstract

Objective: This review aims to provide a summary of the clinical indications and limitations of PET imaging with different radiotracers, including 18F-fluorodeoxyglucose (18F-FDG) and other radiopharmaceuticals, in pediatric neuro-oncology, discussing both supratentorial and infratentorial tumors, based on recent literature (from 2010 to present). Methods: A literature search of the PubMed/MEDLINE database was carried out searching for articles on the use of PET in pediatric brain tumors. The search was updated until December 2020 and limited to original studies published in English after 1 January 2010. Results: 18F-FDG PET continues to be successfully employed in different settings in pediatric neuro-oncology, including diagnosis, grading and delineation of the target for stereotactic biopsy, estimation of prognosis, evaluation of recurrence, treatment planning and assessment of treatment response. Nevertheless, non-18F-FDG tracers, especially amino acid analogues seem to show a better performance in each clinical setting. Conclusions: PET imaging adds important information in the diagnostic work-up of pediatric brain tumors. International or national multicentric studies are encouraged in order to collect larger amount of data.

## 1. Introduction

Central nervous system (CNS) tumors constitute a heterogeneous group of benign and malignant neoplasms with an incidence of 2.99 cases per 100,000 children in Europe [1]. Primary CNS tumors are divided according to their tissue of origin into glial and non-glial tumors. Gliomas constitute the most frequent group of brain tumors in the pediatric population, with pilocytic astrocytoma and brain glioma representing the most common type, with an incidence of approximately 17% and 10%, respectively [1,2].

**Figure 1:**
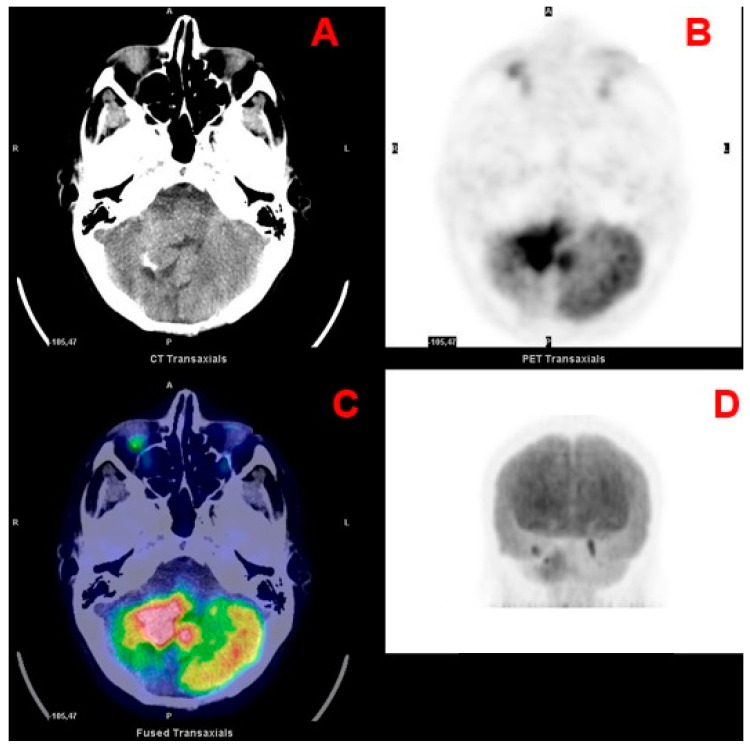
Transaxial CT (**A**), PET (**B**) and fused PET/CT (**C**) and maximum intensity projection (**D**) images of a medulloblastoma.

**Figure 2:**
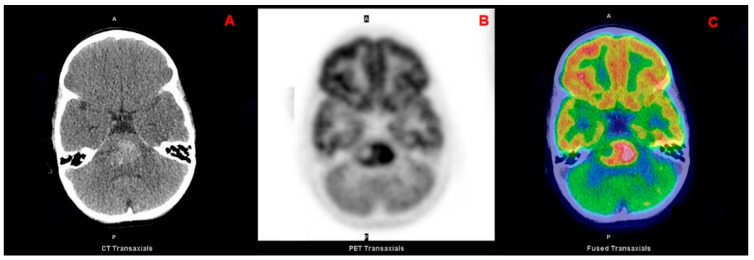
Transaxial CT (**A**), PET (**B**) and fused PET/CT (**C**) images of a pontine glioma.

**Figure 3:**
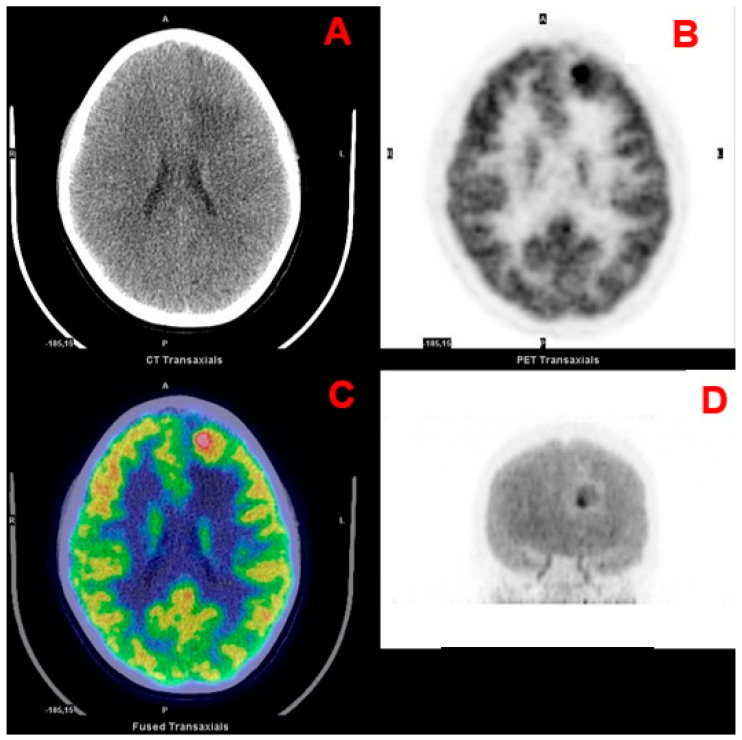
Transaxial CT (**A**), PET (**B**) and fused PET/CT (**C**) and maximum intensity projection (**D**) images of a frontal ganglioglioma.

Another important distinction for pediatric brain tumors, which also influences the surgical approach, is based on the location with respect to the tentorium (supra- and infratentorial) [3]. Differently from adults, most pediatric tumors are infratentorial (approximately 60% of all CNS tumors) and encompass juvenile pilocytic astrocytoma (JPA), medulloblastoma (Figure 1), ependymoma, diffuse intrinsic pontine glioma (DIPG; Figure 2) and atypical teratoid rhabdoid tumor (ATRT) [4,5]. Supratentorial tumors are located in the cerebral hemispheres and are more common in the youngest patients; they include astrocytomas, gangliogliomas (Figure 3), craniopharyngiomas, supratentorial primitive neuroectodermal tumors (PNET), germ cell tumors, dysembryoplastic neuroepithelial tumors (DNET), oligodendrogliomas, and meningiomas [4].

Treatment choice depends on the type of tumor, location, stage, age and clinical conditions, preferring local surgery to remove the primary lesion [6]. The mainstay of initial diagnostic and pre-surgical workup is based on contrast-enhanced magnetic resonance imaging (MRI) with gradient echo, standard T2-weighted, T2-fluid-attenuated inversion recovery (FLAIR), T1-weighted and T1-weighted contrast-enhanced sequences [7]. Although having high anatomical resolution, conventional MRI techniques may not be able to accurately define the extent of a tumor and distinguish tumor and surrounding infiltration from normal tissue, especially in the case of non-focal pediatric brain tumors, such as optic pathway gliomas and brainstem gliomas [8]. The main limitation of conventional MRI is the lack of specificity of the signal abnormality either in T2 weighted imaging (influenced by tissue water content), or gadolinium-contrast enhancement, reflecting vascular surface area and non-specific increased permeability of the disrupted blood-brain barrier (BBB) [9,10]. Other goals of imaging in pediatric brain tumors comprise differential diagnosis of specific tumor types, grading, guiding stereotactic biopsy, differentiation of viable tumor from necrotic tissue, and evaluation of treatment response [11]. As conventional MRI has limited ability in these settings, in many institutions the MRI acquisition protocol includes also advanced MRI techniques, such as diffusion-weighted imaging (DWI), perfusion imaging, MR spectroscopy (MRS), diffusion tensor imaging (DTI), and susceptibility-weighted imaging (SWI) [7,11]. Additionally, low-grade tumors are more common in pediatric patients compared to adults and demonstrate biological and clinical features that are different from low-grade gliomas occurring in adults [1,11,12]. Low-grade tumors do not show significant contrast-enhancement at MRI; furthermore, children are more sensitive to late toxic effects of the tumor and treatment [12,13,14]. Beyond advanced MRI techniques, molecular imaging, and especially positron-emitting tomography (PET), appears to be of paramount importance because may provide additional insights on biological processes such as the assessment of proliferation, glucose metabolism, and uptake of amino acid analogs. These information may be useful for noninvasive grading, differential diagnosis, delineation of tumor extent, surgical and radiotherapy treatment planning, and prognostic stratification [14]. Molecular imaging may also provide an early indication of response to therapy, with unique information on timely biological changes that occur before anatomical changes [15]. Another setting for which molecular imaging may be beneficial is post-treatment surveillance such as pseudoprogression is particularly challenging for MRI [9]. Accordingly, the use of amino acidic PET radiotracers, characterized by high tumor/non-tumor contrast, is increasing in neuro-oncology; however, the available literature involving pediatric patients is still limited, so far. In this scenario, 18F-fluorodeoxyglucose (18F-FDG) plays a fundamental role in pediatrics patients, due to its widespread in the clinical setting despite the limitations of high uptake in normal grey matter and inflammatory lesions (Table 1 and Table 2) [9,15,16].

This review aims to provide a summary of the clinical indications and limitations of PET imaging with different radiotracers (18F-FDG and other radiopharmaceuticals) in pediatric neuro-oncology, discussing both supratentorial and infratentorial tumors, based on recent literature (from 2010 to present).

## 2. Materials and Methods

A literature search of the PubMed/MEDLINE database was carried out searching for articles on the use of PET in pediatric brain tumors. The search was updated until December 2020. The search was limited to original studies published in English after 1 January 2010 and conducted in humans. The references of the retrieved articles were checked to retrieve further relevant studies. 

### Study Selection and Data Extraction

Original articles not in the field of interest of this review, and non-original articles were excluded. The following inclusion criteria were applied to the retrieved original articles: (a) evaluation of the role of PET in pediatric patients (or including a subset of pediatric patients) with known or suspected brain tumor (b) a minimum sample of 10 patients (to minimize the publication bias). Three researchers independently reviewed the titles and the abstracts of the retrieved entries, selecting relevant articles according to the inclusion criteria mentioned above. The full text was retrieved for each selected study and, the main findings of the articles were summarized in the results section according to clinically relevant questions, discussed separately for 18F-FDG and non-FDG tracers.

## 3. Results

### 3.1. Diagnosis, Grading and Delineation of the Target for Stereotactic Biopsy

#### 3.1.1. 18F-FDG 

Few authors illustrated the usefulness of 18F-FDG-PET imaging in supratentorial tumors in the differential diagnosis of brain tumors in pediatric patients. Moharir et al. carried out 18F-FDG-PET scanning and graded tumors in 18 children with optic pathway glioma (OPG; *n* = 19 lesions) or plexiform neurofibromas (PNFs; *n* = 16 lesions) based on 18F-FDG avidity [grade 1: SUVmax < 3 (low), grade 2: SUVmax = 3–4 (intermediate), grade 3: SUVmax > 4 (intense)]. All OPGs graded 1 were asymptomatic, whereas sensitivity (SS) and specificity (SP) of the grading system (intermediate or intense uptake) for detecting symptomatic OPGs were 62.5% and 87.5%, respectively. Furthermore, the authors reported a SS and SP of 100% and 85.7%, respectively, in detecting malignant transformation in children with PNF [18]. 

Another area in which 18F-FDG PET may be clinical useful in targeting of stereotactic biopsy in children with supratentorial tumors. In their experience, Pirotte et al. used 18F-FDG or 11C-methionine (11C-MET), along with MRI, in order to delineate the stereotactic target for biopsy in a mixed group of pediatric brain tumors (15 supratentorial and 20 infratentorial). While MRI provided non-diagnostic tissue samples in 7 (5 supra- and 2 infratentorial tumors) out of 23 MRI-guided biopsies, 18F-FDG or 11C-MET PET imaging guided the collection of a diagnostic tissue sample in all 35 performed biopsies and lead an improved diagnostic yield. In particular, 18F-FDG PET-guided biopsies seems more accurate to differentiate a higher grading than MRI-based guidance in 6 (4 supra- and 2 infratentorial tumors) out of 11 comparisons (PET vs. MRI) and 11C-MET PET allowed the diagnosis of a higher grading than MRI in 8 out of 23 comparisons (7 supratentorial tumor and 1 infratentorial tumor). In a further group of patients (50 resectable tumors: 38 supratentorial and 12 infratentorial lesions), in the same study, the authors carried out 18F-FDG and/or 11C-MET for planning a hypothetical PET-guided volumetric resection and evaluated the potential impact of PET on the planned MRI-based resection. Of note, comparing the tissue samples taken from surgical margins with the post-operative MRI, Pirotte and coworkers did not find tumor tissue left in 37 patients in which no radiotracer uptake was present in the surgical margins. On the other hand, in 12 out of 13 cases, where a residual PET tracer uptake was present in the surgical margins, tumor tissue was still observed at the post-operative MRI [19].

#### 3.1.2. Non-FDG Tracers

Few authors also investigated the utility of non-FDG tracers in supratentorial tumors (Table 3). In 2013, the diagnostic value of 11C-MET PET was demonstrated by Laser et al. in a limited series of ten pediatric patients affected by craniopharyngioma undergoing to PET scanning prior to proton therapy. The authors showed a higher 11C-MET MET tumor uptake to background (white matter) ratio, compared to brain 18F-FDG PET in chraniopharyngioma [23]. 

In a large clinical series of 65 patients with histologically confirmed brain tumors, Laukamp et al. showed that PET imaging is helpful in defining the extent of the tumor and grading. In their article, tumor size, as defined by thresholding based on tumor-to-background ratios (TBRs), was significantly different as measured by 11C-MET PET (21.6 + 36.8 cm^3^), and FLAIR/T2-MRI (64.8 + 60.4 cm^3^; *p* < 0.001), and T1w-Gd-MRI (3.9 + 7.8 cm^3^). A binary logistic regression model differentiated between WHO tumor types with an AC of 80.8% in patients at primary diagnosis [27]. 11C-MET PET appears also useful to differentiate tumors from non neoplastic lesions. A Korean group, in 2010, demonstrated that 11C-MET PET imaging might be clinically useful for the differential diagnosis of lesional epilepsy, in patients with focal cortical dysplasia (FCD), dysembryoplastic neuroepithelial tumor (DNT) and ganglioglioma on the base of lesion-to gray matter (LGR): 1.078 ± 0.182 in 9 evaluated cases of FCD, 1.564 ± 0.368 for DNT (*n* = 5), and 2.114 ± 0.723 for gangliogliomas (*n* = 6) [17]. In another study, including a group of 77 pediatric patients, a negative 11C-MET PET scan successfully differentiated dysembryoplastic neuroepithelial tumors (DNTs) from other epileptogenic brain neoplasms based on visual findings: normal methionine uptake in DNTs (*n* = 21), moderate or marked tumor uptake in all LLGs (*n* = 19) or ganglioma (*n* = 10) [28]. 

Other authors investigated the usefulness of 18F-fluoroethyl-L-tyrosine (FET) [29,30]. Dunkl et al. evaluated this radiocompound for the assessment of newly diagnosed cerebral lesions. In 26 pediatric patients with newly diagnosed brain lesions, the highest AC (77%) to detect tumor tissue (19/26 patients) was achieved when the maximum tumor-to-background-ratio (TBR) was 1.7 or above (area under the curve (AUC) = 0.80 ± 0.09; SS = 79%; SP = 71%; positive predictive value (PPV) = 88%) [29]. Misch et al., instead, demonstrated the potential additional value of 18F-FET PET for targeting the site of biopsy in 26 pretreated patients (age: 12 ± 6.6 years), including various brain tumors (25 WHO grade: I-IV; 1 benign glioneuronal tumor). Tumor was correctly detected by 18F-FET-PET in 20 out of 24 evaluable patients with brain neoplasms based on tumor uptake, using histological examination as reference standard, and a found false positive results was in only two pretreated patients (*n* = 1 lymphocytic tissue, 1 granulation tissue) [30].

Finally, Morana et al. focused on the use 18F-3,4-Dihydroxyphenylalanine (18F-DOPA). The authors assessed the diagnostic value of fusing PET images with conventional MRI images, in a prospective study with a total of 13 pediatric patients, including 11 newly diagnosed supratentorial infiltrative astrocytic tumors, evaluated either before (*n* = 5) of after biopsy (6), and 2 patients with suspected diseased progression after treatment with temozolomide and RT. The authors observed that 18F-DOPA presented a heterogeneous distribution in all positive scans (9/13), in keeping with the heterogeneous tumor habitat, and significantly higher uptake in high-grade tumors compared to low-grade lesions (*p* < 0.05) [31]. 18F-DOPA PET imaging has been found to also detect striatal involvement in patients with pediatric glioma in a retrospective study including 28 children, using 18F-DOPA PET/CT and fused 18F-DOPA PET/MRI in spite of the physiological 18F-DOPA uptake in the striatum [32]. Later, in a retrospective study, Morana and coworkers assessed 26 pediatric patients with diffuse astrocytic tumors using DWI and arterial spin labelling (ASL) MRI sequences, and 18F-DOPA PET, revealing a significant correlation between cerebral blood flow max (rCBF max), DWI-derived minimum apparent diffusion coefficient (rADC min), and 18F–DOPA PET uptake (*p* < 0.001). Additionally in this study, significant difference in 18F-DOPA uptakes were observed between low- and high-grade tumors [33]. 18F-DOPA PET and MRS were evaluated in a further study with twenty-seven patients with supratentorial infiltrative gliomas and demonstrated a direct correlation. The SS, SP, and AC of 18F–DOPA PET for differentiation of gliomas from non-neoplastic lesions were 76%, 83%, and 78%, respectively, and were not significantly different (*p* > 0.05) from those of MRS (SS = 95%, SP = 83%, AC = 93%). 18F–DOPA uptake and 1H-MRS ratios were significantly higher in high-grade lesions than in low-grade tumors (*p* ≤ 0.001 and *p* ≤ 0.04, respectively) [34]. Although with the limit of the small number of patients, the higher uptake of 18F-DOPA in high grade tumors than in low-grade lesions may reflect additional factors other than the expression of amino-acid transporters, namely tumor and aggressiveness (e.g., ki-67 proliferation and tumor grade) and correlation with outcome. Furthermore, since treatment of low-grade gliomas was heterogenous, it is not possible to exclude that treatment influenced 18F-DOPA uptake.

**Table 3:** Major findings of studies on non-FDG PET tracers.

Author	Journal	Year	Tacer	N	Major Findings
Pirotte, B.J. [19]	Journal of neurosurgery. Pediatrics	2010	11C-MET	85	11C-MET PET allowed the diagnosis of a higher grading than MRI
Laser, B.S. [23]	Neuro-oncology	2013	11C-MET	10	(11)C MET PET uptake is significantly greater within the tumor compared with non-involved background white matter, making it more useful than FDG PET in identifying active tumor in patients with craniopharyngioma.
Laukam, K.R. [27]	Mol Imaging	2017	11C-MET	65	Combined PET and MRI improve the evaluation of tumor activity, extent, type/grade prediction, and therapy-induced changes in patients with glioma and serve information highly relevant for diagnosis and management.
Phi, J.H. [17]	J Nucl Med	2010	11C-MET AND 18F-FDG	30	(18)F-FDG does not contribute to the differential diagnosis and that another tracer such as (11)C-methinine is required.
Rheims, S. [28]	Neuro-oncology	2014	11C-MET	77	Normal MET-PET findings in patients with an epileptogenic non-rapidly progressing brain tumor are highly suggestive of DNT, whereas a markedly increased tumor methionine uptake makes this diagnosis unlikely.
Misch, M. [30]	ChNS: official journal of the International Society for Pediatric Neurosurgery	2015	18F-FET	26	(18)F-FET-PET imaging is helpful for target selection and can be integrated in surgical guidance. (18)F-FET-PET image-guided surgical targeting yielded histological diagnosis in pediatric brain tumor patients.
Morana, G. [31]	J Nucl Med	2014	18F-DOPA	13	(18)F-DOPA PET/MR image fusion may be a reliable imaging biomarker of pediatric IAs. Information gathered by this combined imaging approach can be readily transferred to the everyday practice and may help clinicians to better stratify patients with IAs, especially diffuse astrocytomas and gliomatosis cerebri, for diagnostic, therapeutic, and prognostic purposes.
Morana, G. [32]	European journal of nuclear medicine and molecular imaging	2016	18F-DOPA	28	(18)F-DOPA PET/CT correctly detected involvement of the dorsal striatum in lesions with a T/S ratio >1, but appeared to be less suitable for evaluation of the ventral striatum. The use of fused (18)F-DOPA PET/MRI further improves the accuracy for evaluation of the ventral striatum.
Morana, G. [33]	European journal of nuclear medicine and molecular imaging	2017	18F-DOPA	26	18F-DOPA PET provide useful complementary information for pediatric DAT grading. 18F-DOPA uptake better correlates with PFS prediction. Combining MRI and PET data provides the highest predictive power for prognosticating tumor progression
Morana, G. [34]	Neuro-oncology	2015	18F-DOPA	27	(18)F-DOPA uptake better discriminates low-grade from high-grade gliomas and is an independent predictor of outcome vs H-MRS
Marner, L. [35]	Clin Transl Imaging	2017	18F-FET	300	PET/MRI scan may increase accuracy in discriminating recurrence from treatment changes, although sequential same-day imaging on separate systems will often constitute a reliable and cost-effective alternative.

### 3.2. Prognosis

#### 3.2.1. 18F-FDG

There is evidence supporting that higher 18F-FDG uptake is directly correlated with higher histologic grade and provides prognostic information in pediatric patients with either supra- or infra-tentorial brain tumors [20,22,24]. Interestingly, an analysis of a large cohort of pediatric patients (*n* = 203) with supra- and infra-tentorial newly diagnosed (*n* = 66) and recurrent/refractory brain tumors (*n* = 137) demonstrated various 18F-FDG and MRI metabolic patterns according to the tumor type. In patients (*n* = 53) with newly diagnosed brain stem gliomas (BSG), the presence of correlation between contrast-enhancement and the distribution of 18F-FDG uptake in the tumor lesion was significantly associated with longer OS (*p* = 0.032). Instead, in refractory/recurrent gliomas, the lack of correlation between 18F-FDG uptake distribution and contrast-enhancement was associated with a better progression-free survival (PFS) (*p* = 0.023) [24].

Recently, in a prospective study, Goda et al. evaluated the prognostic value of preoperative multiparametric MRI (mMRI) and 18F-FDG-PET/CT in 20 infratentorial tumors, namely newly diagnosed diffuse intrinsic pontine glioma (DIPG) [21]. Considering different mMRI parameters (i.e., contrast-enhancement, perfusion, spectroscopy, etc.), the authors built a radiological prognostic cumulative index (RPI) which resulted able to divide patients in low-grade (5/20, LGG), intermediate-grade (3/20) and high-grade (12/20) glioma (HGG). Additionally, the RPI index resulted able to significantly stratify overall-survival (OS) according to their grade (*p* = 0.02). For 18F-FDG-PET the authors observed an inverse correlation between uptake and survival; indeed, patients with increased FDG uptake had lower OS and PFS compared to patients with lower FDG uptake (40% and 33% versus 66.7% and 40%, respectively). In terms of accuracy (AC), the best results were obtained in HGGs as by perfusion and spectroscopy MRI (SS and SP = 100%), as for 18F-FDG-PET (SS = 100%; SP = not available). Lower values were obtained for both modalities in LGGs. Discordant results were reported by Zukotynski et al. [20] for infratentorial tumors, namely in 40 children affected by brain stem glioma (BSG), who underwent baseline 18F-FDG PET/CT before radiation therapy (RT) plus molecular targeted agents (tipifarnib or gefitinib) and were analyzed using different PET/CT features, such as intensity and uniformity of uptake. The intensity of tracer uptake in glioma was stratified using a 5-point scale score (1 = no uptake; 2 = uptake similar to normal white matter; 3 = uptake between normal white and gray matter; 4 = uptake similar to normal gray matter; and 5 = uptake greater than normal gray matter). Uniformity uptake of primary tumor was defined as the percentage of the tumor (using FLAIR MR images) with increased 18F-FDG uptake and was ranked on a 4-point scale (0 ≤ 25%; 1 = 25%–50%; 2 = 51%–75%; and 3 ≥ 75%). They demonstrated that intensity of uptake was not correlated with PFS (*p* = 0.36) and OS (*p* = 0.48); instead, considering uniformity, patients with more than half of tumor with increased FDG uptake had significantly shorter PFS (*p* = 0.031) and OS (*p* = 0.086). A further recent paper by Zukotynski et al. [26] focused on DIPG supporting the utility of integrating MRI with apparent diffusion coefficient (ADC) with 18FDG PET imaging. The authors evaluated the potential prognostic role of textural parameters (skewness or kurtosis) of 18F-FDG uptake. Despite not significant correlation of skewness or kurtosis with PFS, a higher post gadolinium FDG histogram skewness tended towards a less favorable PFS (HR = 3.48; *p* = 0.11). Furthermore, the degree of correlation between PET-derived parameters and ADC was significantly correlated with PFS; glioma with higher values of ADC-PET correlation had more favorable PFS (hazard ratio = 0.17 *p* = 0.036).

#### 3.2.2. Non-FDG Tracers

Some evidence exists on the usefulness of 18F-DOPA PET in outcome prediction in pediatric patients with astrocytic tumors. Morana et al. first, provided evidence of correlation of 18F-DOPA uptake with PFS in a group of 13 pediatric patients with different types of supratentorial infiltrative astrocytoma (*p* < 0.04) [31]. These finding are in keeping with a larger, retrospective, study with 27 pediatric patients with supratentorial infiltrative brain lesions, in which 18F-DOPA uptake correlated with PFS (*p* ≤ 0.05) and OS (*p* = 0.04) [34]. The same group also evaluated twenty-six patients with astrocytic tumors confirming a good performance of 18F-DOPA PET (AUC = 0.93, *p* < 0.001) in predicting tumor progression. An increase in the prognostic ability was achieved combining PET and MRI data obtained from DWI and ASL (AUC = 0.93, *p* < 0.001) [33]. Finally, Rosenfeld et al. calculated the survival curve of 25 pediatric patients with diffuse intrinsic brainstem gliomas (DIBSG), based on 18F–FDG and 11C-MET tumor uptake and suggested the combined use of these radiotracers [36]. Patients with both 18F-FDG and 11C-MET positive PET scans had a mean survival of 380 days, while patients with both negative 18F–FDG and 11C-MET PET scans had a mean survival of 446 days. Patients with the shortest survival time were those that presented 18F–FDG negative and 11C-MET positive scans (*n* = 2; 229 days), suggesting presence of extensive tumor necrosis and an aggressive nature of the tumor. Both negative 18F–FDG and 11C-MET scans represented the metabolic pattern with longer survival, presumably mirroring low-grade histopathology.

### 3.3. Evaluation of Recurrence

#### 3.3.1. 18F-FDG

Distinguishing recurrence from non-tumoral lesions after RT and/or chemotherapy is a crucial problem. Radionecrosis (RN) constitutes a severe long-term complication. Its diagnosis is challenging, as MRI cannot clearly distinguish recurrence from RN. A retrospective single-center study in a cohort of 107 children treated with external radiotherapy for different brain tumors found an incidence of 4.7% of RN substantiated by 18F-FDG [37].

So far, no papers investigated the usefulness of 18F-FDG PET/CT in the assessment of recurrence in selected groups of pediatric patients with supratentorial tumors. Only a case series of 5 pediatric patients studied the potential role of 18F-FDG PET/CT in discriminating between RN and recurrence and showed a reduction of 18F-FDG uptake in 3 patients with RN [37]. No other studies analyzed the performance of 18F-FDG PET imaging in this setting in the last 10 years probably due the high AC of amino acid tracers in this setting both for supra- and intra-tentorial tumors.

#### 3.3.2. Non-FDG Tracers

The literature regarding the role of PET with amino acid tracers in the evaluation of recurrence appears more promising compared to 18F-FDG. In a study of Marner et al. detection rate of 18F-FET-PET and MRI were assessed in a cohort of twenty-two patients. 18F-FET-PET combined with MRI discriminated tumor from treatment effects with a lesion-based SS, SP, and AC of 73%, 100% and 87%, whereas SS, SP and AC of MRI alone were 80%, 75%, and 77% [35]. Dunkl et al. evaluated the usefulness of dynamic 18F-FET PET in the clinical evaluation of 48 pediatric patients with brain tumors. A high AC (82%) for detection of brain tumor progression or recurrence was obtained according to specifical patterns of peak uptake after injection and subsequent plateau (AUC = 0.80 ± 0.11; SS = 75%; SP = 90%) [29].

### 3.4. Treatment Planning and Assessment of Treatment Response

#### 3.4.1. 18F-FDG

A major issue in treating pediatric brain tumors is the limited ability to predict and monitor response to therapy, particularly early after initiating therapy. MRI is not an effective method for detection of early response. 18F-FDG PET/CT may help in evaluation of treatment response in patients with supratentorial brain tumors [18,21,25]. Mohair et al. stratified a cohort of 18 children with various supratentorial tumors, namely OPG or plexiform neurofibroma (PNF), according to the maximum standardized uptake value (SUV max) of the tumor into (a) grade 1 if SUVmax < 3; (b) grade 2 for SUVmax between 3–4; (c) grade 3 for SUVmax > 4. Ten patients were grade 1, three were grade 2 and two were grade 3. A reduction of 18F-FDG uptake was noted in 2 patients with OPG showing a shift from grade 3 to 1 after chemotherapy, associated also with clinical improvement. A direct correlation was also observed between SUVmax and severity of clinical symptoms. Furthermore, the authors showed that 18F-FDG-avid OPGs are more likely to become symptomatic than non 18F-FDG-avid ones, suggesting 18F-FDG as a useful marker of malignant potential and proliferation for the assessment of risk of progression [18]. Nevertheless, imaging response assessment by 18F-FDG PET seems not to be related with OS as showed by Goda et al. in 11 patients with DIPG, who performed 18F-FDG PET PET/CT after first line of therapy although this finding may have be biased from the limited statistical power of the patient sample [21].

#### 3.4.2. Non-FDG Tracers

The use of PET imaging with radiolabeled amino acids targeting L-amino acid transporter system is widely recommended in adult neuro-oncology. Recent guidelines by the Response Assessment in Neuro-Oncology (RANO) working group and European Association for Neuro-Oncology state that amino acid tracers overcome to 18F-FDG in distinguishing neoplastic from non-neoplastic tissue and may be complementary to MRI [9]. The limited available literature suggest that radiolabeled amino acids may have similar utility in pediatric patients.

Apart from amino acid tracers, choline-based radiotracers have also been used in the scenario of the pediatric brain tumors. These tracers were initially developed to image prostate cancer and mirror increased cell membrane synthesis and proliferation [38]. Fraioli et al. examined functional MRI and 18F-fluoroethylcholine (18F-choline) PET images for diagnosis and assessment of response to therapy in astrocytic brain tumors in a cohort of twelve pediatric patients (8/12 undergoing also additional scans after treatment) evaluated by means of a hybrid PET/MRI scanner. The authors observed that in four out of eight patients, although not reaching a statistical significant difference, a concordant decrease in mean size of the tumor (from 2.3 to 2 cm) and SUVmax/mean and an increase of ADC in three of these patients, suggesting a correlation between high cellularity and metabolic activity [21,39]. The same group, one year later, demonstrated the potential role of 18F-Choline PET/MRI in detecting viable residual tumor cells in a series of four patients with proven or suspected intracranial non-germinomatous germ cell tumors. Indeed, the authors reported in the post-therapy (neoadjuvant chemo-radiotherapy or radiotherapy) scans of two patients persistent 18F-Choline uptake, in keeping with viable tumor as confirmed by the post-surgery histological examination. On the contrary, no tracer uptake was demonstrated in two patients with negative histology after treatment [40].

Treatment for pediatric brain tumors may also include bevacizumab, a humanized anti-VEGF monoclonal neutralizing antibody [36]. Gauvain and colleagues demonstrated the safety and feasibility of assessing early response (at 3 months after initiating therapy) in keeping with subsequent clinical response to treatment in six pediatric patients with recurrent gliomas (five low-grade, one high-grade) by means of changes in MTV using an hybrid 18F-FDOPA PET/MRI scanner [41].

## 4. Discussion/Future Perspectives

In the diagnostic field of pediatric neuro-oncology, a fully integrated PET/MRI scanner reduce radiation exposure compared to PET/CT scans and may offer co-registered multimodal, high-resolution data for neuronavigation, intended as computer-assisted technologies integrating imaging modality on neurosurgery assessment. Therefore, one single scan would be able to provide both morphological and functional data for neuronavigation and preoperative planning, therefore avoiding additional anesthesia in the small patients [37]. Similarly, PET/MRI with the combination of MRI and radiolabeled amino-acid analogs may offer complementary information in a single exam, in order to exactly estimate the true tumor extent both in low- and high-grade gliomas. Regarding such fully integrated systems, a considerable issue could be the choice of proper MR-based sequences for attenuation correction. Ladefoged et al. recently published the results of a study on simultaneous 18F-FET-PET/MRI using deep learning-based attenuation correction in 79 pediatric patients with brain tumor. The use of artificial intelligence applied to MRI sequences seemed to be able to improve the quality of PET/MRI imaging [35].

Recent studies have also explored the application of new experimental radiotracers. Just one evidence reported the feasibility and tumor detection of 68Ga-NOTA-Aca-BBN(7-14) PET and MRI in OPG. In a small cohort of children, tumor-to-background ratio, SUVmax and SUVmean were found significantly higher for 68Ga-NOTA-Aca-BBN(7-14) than for 18F-FDG [42]. Another study investigated the utility of 89Zr-bevacizumab [43]. PET/CT images, acquired 1, 72 and 144 h after the administration of 0.9 MBq/kg-0.1 mg/kg of 89Zr-bevacizumab in 7 pediatric patients with DIPG. Interestingly, images showed a significant uptake (SUV range = 1–6.7 at 144 h p.i) even in tumor areas without MRI contrast-enhancement.

Pharmacokinetics and biodistribution of PET radiopharmaceuticals greatly influence their ability for imaging brain tumors, therefore an overall knowledge of their main features is of paramount importance for the choice of the most appropriate radiotracer based on the brain tumor to investigate [44]. 18F-FDG readily crosses the BBB and its accumulation in tumor cells is mainly regulated by expression of the glucose transporter protein (GLUT) and the action of hexokinase. Nevertheless, the high uptake 18F-FDG in normal grey matter has limited its use in the vast majority of low-grade tumors Uptake of amino-acid tracers is independent of BBB breakdown and show low retention in normal grey matter [45]. Their uptake in tumor cells reflect the level of amino-acid transport. Still, some low-grade tumors may show increased amino acid uptake due to augmented vascularity and/or upregulation of amino acid transporters at the BBB [46].

## 5. Conclusions

Literature published in the last 10 years focusing on PET imaging in pediatric brain tumors appears fragmentary. 18F-FDG PET imaging appears useful in several clinical settings in pediatric neuro-oncology, and especially for grading, predicting malignant transformation and detecting the most representative bioptic site of tumor aggressiveness. Furthermore, 18F-FDG PET may be considered as a beneficial supplement to MRI for prognostic stratification of patients, evaluation of recurrence and assessment of response to therapy. Non-FDG tracers may provide a higher performance in each clinical scenario. Future studies may stratify patients based on risk factors, such as genetic, MRI features, in order to shed light in additional diagnostic and prognostic information. Furthermore, in order to collect a large amount of data, international or national multicentric studies are encouraged.

## Figures and Tables

**Table 1:** Biographical features of the main studies using 18F-FDG PET imaging in pediatric brain tumors.

1st Authors	Year	Journal	Country	Study Design	N pts	Tumor Location	Histology
Phi [17]	2010	JNM	Korea	retrospective	30	supratentorial	11 FCD; 8 DNT; 11 GG
Moharir [18]	2010	EJNMMI	Australia	retrospective	18	supratentorial	7 OPG; 7 PNF; 4 OPG + PNF
Pirotte [19]	2010	J neurosurg pediatrics	Belgium	prospective	85	Supratentorial infratentorial	10 GBM; 10 AA; 13 LGA; 5 PNET; 3 germ cell tumor; 14 PA; 11 ependymoma; 9 GG; 10 OD
Zukotynski [20]	2011	JNM	USA	prospective	40	NR	NR
Goda [21]	2013	Ped Neurosurgery	India	prospective	20	infratentorial	20 DIPG
Zukotynski [22]	2013	JNM	USA	retrospective	24	supratentorial infratentorial	7 HGG; 9 LGG; 4 BSG; 2 medulloblastoma; 2 ependynoma
Laser [23]	2013	Neuro-oncology	USA	prospective	10	supratentorial	craniopharyngioma
Zukotynski [24]	2014	JNM	USA	retrospective	203	supratentorial infratentorial	71 BSG; 24 GBM; 30 AA; 23 astrocytoma; 15 ependymoma; 10 medulloblastoma; 5 pineoblastoma; 25 other
Hua [25]	2015	JNS pediatrics	USA	prospective	50	supratentorial	craniopharyngioma
Zukotynski [26]	2017	JNM	USA	prospective	33	infratentorial	33 PG

FCD: focalcorticaldysplasia; DNT: dysembryoplasticneuroepithelial tumors; GG: ganglioglioma; OPG: opticpathwaygliomas; PNF: plexiformneurofibroma; AA: anaplasticastrocytoma; LGA: low-grade astrocytoma; GBM: glioblastoma multiforme; PNET: primitive neuroectodermal tumor; OD: oligodendroglioma; PA: pilocyticastrocytoma; DNET: dysembryoplasticneuroectodermal tumor; DIPG: diffuse intrinsic pontine glioma; HGG: high-grade glioma; BSG: brain stem glioma; NR: not reported.

**Table 2:** Technical features of the main studies using 18F-FDG PET imaging in pediatric brain tumors.

1st Authors	Device	Activity Injected MBq Mean (Range)	Uptake Time Min Mean (Range)	PET Analysis	Semiquantitative Parameters	SUV Max Mean (Range)
Phi [17]	PET or PET/CT	7.4 MBq/Kg	40	visual and semiquantitative	LGR	NR
Moharir [18]	PET/CT	370 MBq	30	visual and semiquantitative	SUVmax	2.89 (1.75–5.57)
Pirotte [19]	PET	222–333	40–60	visual	N/A	N/A
Zukotynski [20]	PET	5.55 MBq/Kg (18–370)	40–60	visual	N/A	N/A
Goda [21]	PET/CT	NR	NR	visual and semiquantitative	SUVmax	NR
Zukotynski [22]	PET	5.55 MBq/Kg (18–370)	40–60	visual	N/A	N/A
Laser [23]	PET/CT	5.5 MBq/Kg	60	visual and semiquantitative	SUVmax	2.65 (1.3–7.4)
Zukotynski [24]	PET	5.55 MBq/Kg (18–370)	40–60	visual	N/A	N/A
Hua [25]	PET/CT	5.55 MBq/Kg (74–444)	60	visual and semiquantitative	SUVmax and ratios	NR
Zukotynski [26]	PET	5.55 MBq/Kg (18–370)	40–60	visual and semiquantitative	SUVmax	NR

LGR: lesion to gray matter ratio; NR: not reported; N/A: not applicable.

## Data Availability

Data is contained within the article.
